# Stereotactic radiosurgery of brain metastases: a retrospective study

**DOI:** 10.1186/s13014-023-02389-z

**Published:** 2023-12-19

**Authors:** Isabella Gruber, Karin Weidner, Marius Treutwein, Oliver Koelbl

**Affiliations:** Department of Radiation Oncology, University Hospital Regensburg, Franz-Josef-Strauss Allee 11, Regensburg, Bavarian 93053 Germany

**Keywords:** Stereotactic radiosurgery, Fractionated stereotactic radiotherapy, Brain metastases, Brain radiation necrosis

## Abstract

**Background:**

Single-fraction stereotactic radiosurgery (SRS) is an established standard for radiation therapy of brain metastases although recent developments indicate that multi-fractionated stereotactic radiotherapy (FSRT) results in lower radiation necrosis especially for larger metastases, and the same or even better local control in comparison to SRS.

**Methods:**

Seventy-two patients with 111 brain metastases received SRS with a single dose of 18 Gy between September 2014 and December 2021. The dose prescription was either 18 Gy given to the enclosing 80% isodose with a normalization to Dmax = 100% of 22.5 Gy (part I) or 18 Gy = D98, while D0.03 cc of 21.6–22.5 Gy was accepted (part II). The study retrospectively evaluated local progression-free survival (LPFS), response on the first follow-up magnetic resonance imaging (MRI), and radiation necrosis.

**Results:**

Melanoma brain metastases (n = 44) were the most frequent metastases. The median gross tumor volume (GTV) was 0.30 cm³ (IQR, 0.17–0.61). The median follow-up time of all patients was 50.8 months (IQR, 30.4–64.6). Median LPFS was 23.5 months (95%CI 17.2, 29.8). The overall LPFS rates at 12-, 18-, 24- and 30 months were 65.3%, 56.3%, 46.5%, and 38.8%. Brain metastases with radioresistant histology (melanoma, renal cell cancer, and sarcoma) showed a 12-month LPFS of 60.2%, whereas brain metastases with other histology had a 12-month LPFS of 70.1%. The response of brain metastases on first follow-up MRIs performed after a median time of 47 days (IQR, 40–63) was crucial for long-term local control and survival. Eight brain metastases (7.2%) developed radiation necrosis after a median time of 18.4 months (IQR, 9.4–26.5). In multivariate analyses, a GTV > 0.3 cm³ negatively affected LPFS (HR 2.229, 95%CI 1.172, 4.239). Melanoma, renal cell cancers, and sarcoma had a lower chance of LPFS in comparison to other cancer types (HR 2.330, 95%CI 1.155, 4.699).

**Conclusions:**

Our results indicate a reasonable 1-year local control of brain metastases with radiosensitive histology. Radioresistant metastases show a comparatively poor local control. Treatment refinements merit exploration to improve local control of brain metastases.

**Trial registration:**

This study is retrospectively registered (ethics approval number 23-3451-104).

## Background

Single-fraction stereotactic radiosurgery (SRS) without additional whole-brain radiotherapy (WBRT) is a national and international standard for local radiotherapy of limited brain metastases (1–4 brain metastases) [1, 2]. SRS is advised even in patients with 5–10 brain metastases [3]. At this point, SRS is the only and first modality of stereotactic radiotherapy analyzed in prospective and randomized studies [4, 5]. The Radiation Therapy Oncology Group (RTOG) protocol 90 − 05 recommends SRS for brain metastases up to 4.0 cm in maximum diameter. However, the risk of radiation necrosis increases with the size of the metastases in cases without dose reduction [4]. Data indicate that the maximum tolerated dose is 24 Gy for brain metastases ≤ 2.0 cm, 18 Gy for metastases of 2.1 to 3.0 cm, and 15 Gy for metastases of 3.1 to 4.0 cm [4]. Multi-fractionated stereotactic radiotherapy (FSRT) delivering the total dose over several fractions plays a minor role in guidelines as prospective and randomized studies comparing the efficacy of FSRT and SRS are lacking. In recent years, FSRT has been used as an alternative to SRS in large-sized brain metastases and in metastases with contact with sensitive organs at risk (e.g., optic nerves, brain stem) [6, 7]. FSRT is especially favored based on radiobiological considerations suggesting a promise in local control due to the 4 Rs of tumor cells (re-oxygenation, repair, repopulation, and redistribution) between the fractions [8, 9]. However, the role of the 4 Rs and the linear-quadratic (LQ) model may be limited in single-fraction SRS known to cause additional cell death through indirect mechanisms (e.g., vascular damage, anti-tumor immune response) [10].

Tumor size [11, 12] and SRS doses [9] are prognostic factors for local progression after SRS, affecting the generalizability of the results. Retrospective studies of SRS of small brain metastases report 1-year local control rates ranging from 86% (20–24 Gy SRS for metastases ≤ 1 cm diameter [13]) to 56% (18 Gy SRS for brain metastases of a median diameter of 1.0 cm [14]) and the risks of radiation necrosis as high as 15% in these studies [14]. However, different tumor entities with varying radio-sensitivity complicate the interpretation of these results.

Nevertheless, SRS is an established standard for radiation therapy, and convincing data indicate a lower incidence of radiation necrosis with FSRT than with SRS [15, 16]. This retrospective study analyzed local control, response on the first follow-up MRI, and radiation necrosis of brain metastases treated with 18 Gy SRS. To further address local control of different cancer types, we separately analyzed local control of brain metastases with radioresistant and radiosensitive histology. Additionally, we contribute to the existing literature by discussing the results in the context of findings of SRS and FSRT of brain metastases.

## Methods

### Data collection

We retrospectively analyzed local control and radiation necrosis after SRS of brain metastases at the Department of Radiation Oncology of the University Hospital Regensburg, Germany. Eligibility criteria for this retrospective analysis included patients with intact brain metastases of solid cancers who received SRS in one fraction of 18 Gy without concurrent or prior WBRT between September 2014 and December 2021. Prior stereotactic radiotherapy (SRS, FSRT) and resection of the brain metastasis to be analyzed were not allowed. Patients without follow-up magnetic resonance imaging (MRI) at baseline or at least once after SRS were excluded. All patients were reviewed at a multidisciplinary tumor conference accounting for tumor- and patient-related factors. Clinical data were extracted from the medical charts of the University Hospital Regensburg. Variables included patient age at the time of SRS, sex, diagnosis, initial UICC stage according to the TNM Classification of Malignant Tumours (8th edition), date of SRS, gross tumor volume (GTV), planning target volume (PTV), location of brain metastases, control of primary cancer, presence of extracranial metastases, number of brain metastases, Karnofsky performance score (KPS), recursive partitioning analysis (RPA) [17], and diagnosis-specific graded prognostic assessment (ds-GPA) [18]. Patient age and KPS were assessed on the day of SRS. Extracranial disease status and control of primary cancer related to the last medical examination before SRS. Variables related to outcome were local progression-free survival (LPFS), overall survival (OS), distant brain progression-free survival (DPFS), the response of brain metastases on 1st follow-up MRI, and response at the end of the follow-up period. We retrospectively differentiated between radiation necrosis, local progression, and response of brain metastases based on follow-up imaging and/or histology in cases of resection. Data closing was in July 2023. The local Ethics Board of the University of Regensburg approved the analysis (ethics approval number 23-3451-104).

### Stereotactic radiosurgery and response assessment

Patients were immobilized with an individually manufactured stereotactic mask system of thermoplastic material (Brainlab, Munich, Germany). Planning computed tomogram (CT) slice thickness was 1 mm. Baseline diagnostic contrast-enhanced T1-weighted MRIs were co-registered with CT scans. MRIs used were not allowed to be older than 2 weeks. The gross tumor volume (GTV) was delineated in the contrast-enhanced T1 sequence of the MRI. The planning target volume (PTV) was created with a margin of 2 mm. All brain metastases received 18 Gy single-fraction SRS. The dose prescription was either 18 Gy given to the enclosing 80% isodose with a normalization to Dmax = 100% of 22.5 Gy (part I) or 18 Gy = D98, while D0.03 cc of 21.6–22.5 Gy was accepted (part II). This change has been caused by the exchange of the treatment planning system, not allowing the same prescription technically and resulting in slight differences only. First, we used Oncentra® external beam treatment planning system and collapsed cone algorithm for dose calculation from January 2017 to October 2018 (part I), and second Monaco® treatment planning system with Monte Carlo dose calculation from November 2018 to December 2021 (part II). All patients received coplanar and non-coplanar 6 megavoltage (MV) photon beams with a linear accelerator of type Elekta SynergyS™ (Elekta Ltd, Crawley, UK). Daily kV X-ray/cone beam CT imaging was used for daily setup verification and repositioning. The biologically effective dose (BED) was 36 Gy using the LQ model with an alpha/beta of 12 Gy for brain metastases, and the formula of Wiggenraad et al. [9]. Patients were followed up with MRIs at 6–8 weeks after SRS and about every 3 months thereafter until the last follow-up appointment or earlier in the cases of neurological deficits. As in our previous study [19], we used modified definitions for the response assessment of brain metastases similar to proposals from the Response Assessment in Neuro-Oncology Brain Metastases (RANO-BM) group [20]. Complete response and partial response were defined as the disappearance of the irradiated brain metastasis in contrast-enhanced MRI and at least a 30% decrease in the sum longest diameter of the brain metastasis, respectively [20]. Progressive disease was defined as at least a 20% increase in the sum longest diameter of the brain metastasis and an increase by 5 mm or more. Stable disease was defined as neither fulfilling the criteria for progressive disease nor partial response [20]. In cases of patients with > 1 brain metastases, the worst response was analyzed and progression was defined as at least one brain metastasis fulfilling the criteria of progression. In cases of radiographic assumption of progression, but clinical evidence assumes radiologically changes/brain radiation necrosis due to treatment effects and not to progression, MRIs were repeated in a shorter time interval. Advanced imaging (perfusion MRI, PET-CT with amino acids) was not available in each case. The continued growth of the enhancing areas in follow-up imaging was considered as radiographic progression. Stable disease or regression of enhancing areas on serial follow-up MRIs was retrospectively considered as a response. If repeated imaging or pathology showed a response or progression, the date of response or progression was recorded as the date of the initial scan [20].

### Definitions and statistical endpoints

The primary endpoint was LPFS. Secondary endpoints were OS, DPFS, response on 1st follow-up MRI, response at the end of follow-up, and frequencies of brain radiation necrosis. All times to the endpoints were calculated from the day of SRS. LPFS was defined as the time between the day of SRS and the first follow-up MRI showing the in-field progression of the irradiated brain metastasis. DPFS was defined as the time between the day of SRS and distant brain failure (appearance of new or progressive brain metastases outside the PTV). OS was defined as the time from SRS to the date of death by any cause. If a patient was event-free for all of the endpoints, the patient was censored at the last date of MRI or follow-up with confirmation of being event-free. OS, LPFS, and DPFS were evaluated using Kaplan-Meier estimators. Prognostic factors predicting OS and LPFS were analyzed with univariate and multivariable regression analyses. Predicting variables were GTV, extracranial metastases, control of primary cancer, systemic treatment 3 months before/after SRS, KPS, patient age, RPA, dsGPA, histology, cerebral progression outside of the PTV, and number of brain metastases.

### Statistical analysis

Characteristics are presented as median and interquartile range (IQR) for continuous variables and as absolute and relative frequencies for categorical variables. Overall survival was estimated using the Kaplan-Meier method. Median follow-up time was calculated using the reverse Kaplan-Meier method with indicator variables reversed [21]. OS and LPFS were analyzed by univariable and multivariable Cox proportional hazard regression models. Hazard Ratio (HR) and 95% - confidence interval (95% - CI) were presented as effect estimates. All *P*-values were two-sided. *P*-values < 0.05 were considered significant. SPSS statistical software (SPSS Inc., version 26.0, Chicago, IL, USA) was used for statistical analyses.

## Results

### Patient and brain metastasis characteristics

In summary, the study included 111 brain metastases in 72 patients. Seven patients previously treated with WBRT were excluded. Two patients who died before the first follow-up MRI were not evaluated. The median follow-up time of the included patients (n = 72) was 50.8 months (IQR, 30.4–64.6). The median times from cancer diagnosis to SRS of brain metastases were 19.5 months (IQR, 9.5–43.3). Patient characteristics (*n* = 72) and brain metastasis characteristics (n = 111) at the time of SRS are shown in Table 1. The most common primary cancers were malignant melanoma (41.7%). The median GTV of brain metastases was 0.30 cm³ (IQR, 0.17–0.61). In the 1st follow-up MRI performed after a median time of 47 days (IQR, 40–63), 41.4% of brain metastases showed responsive disease, 53.2% stable disease, and 5.4% progressive disease (Table 1).

**Table 1:** Patient characteristics (*n* = 72) and brain metastasis characteristics (n = 111)

Characteristics	Value
Patient characteristics (n = 72)	
Patient age, years, median (interquartile range, IQR)	62 (55–68)
**Sex, n (%)**	
Men	41 (56.9%)
Women	31 (43.1%)
**Primary cancer, n (%)**	
Malignant melanoma	30 (41.7%)
NSCLC adenocarcinoma	21 (29.2%)
NSCLC non-adenocarcinoma	10 (13.9%)
Breast cancer	4 (5.6%)
Gastrointestinal carcinoma	3 (4.2%)
Sarcoma	2 (2.8%)
Renal cell carcinoma	1 (1.4%)
Adrenal gland cancer	1 (1.4%)
**Initial UICC stage, n (%)**	
I	3 (4.2%)
II	10 (13.9%)
III	12 (16.7%)
IV	47 (65.3%)
Karnofsky performance score, median (IQR)	70 (70–80)
Systemic treatment (chemotherapy, immunotherapy, targeted therapy, or anti-hormonal therapy) three months before/after SRS, n (%)	65 (90.3%)
Number of brain metastases treated with 18 Gy SRS, median (IQR)	1.0 (1.0–2.0)
Recursive partitioning analysis (RPA), median (IQR)	2.0 (2.0–3.0)
Diagnosis-specific graded prognostic assessment (ds-GPA), median (IQR)	2.0 (1.5–3.0)
**Disease control of the primary cancer, n (%)**	
Yes	42 (58.3%)
No	30 (41.7%)
**Extracranial metastases, n (%)**	
Yes	43 (59.7%)
No	29 (40.3%)
**Brain metastasis characteristics (n = 111)**	
**Cancer types**	
Malignant melanoma	44 (39.6%)
NSCLC adenocarcinoma	28 (25.2%)
NSCLC non-adenocarcinoma	14 (12.6%)
Breast cancer	8 (7.2%)
Gastrointestinal carcinoma	8 (7.2%)
Sarcoma	6 (5.4%)
Adrenal gland cancer	2 (1.8%)
Renal cell carcinoma	1 (0.9%)
**Location, n (%)**	
Supratentorial	97 (87.4%)
Infratentorial	14 (12.6%)
**Gross tumor volume, GTV (cm³)**	
Mean (Standard deviation, SD)	0.50 (0.51)
Median (IQR)	0.30 (0.17–0.61)
**Planning target volume, PTV (cm³)**	
Mean (SD)	1.91 (1.24)
Median (IQR)	1.57 (0.95–2.44)
**Response of metastases on 1st follow-up MRI after a median time of 47 days (IQR, 40–63), n (%)**	
Responsive disease †	46 (41.4%)
Stable disease ‡	59 (53.2%)
Progressive disease §	6 (5.4%)

*IQR*, interquartile range; *SD*, standard deviation; *GTV*, gross tumor volume; *PTV*, planning target volume; *MRI*, magnetic resonance imaging.

†Complete response (disappearance of the brain metastasis on follow-up MRI) and partial response (at least a 30% decrease in the sum longest diameter of the brain metastasis) were summarized as responsive disease.

‡Stable disease was defined as neither fulfilling the criteria for partial response nor progressive disease.

§Progressive disease was defined as at least a 20% increase in the sum longest diameter of the brain metastasis and an increase by 5 mm or more.

## Overall survival

**Fig. 1:**
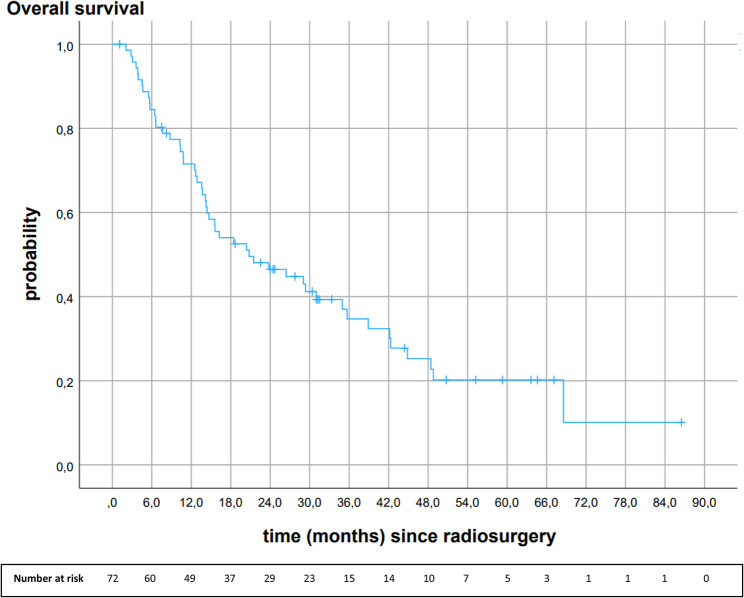
Kaplan-Meier analysis of overall survival after 18 Gy stereotactic radiosurgery (*n* = 72)

Median OS was 20.8 months (95%CI 8.9, 32.7). Overall survival rates at 6-, 12-, 18-, 24-, and 30 months were 84.5%, 71.5%, 54.0%, 46.5%, and 41.2%, respectively (Fig. 1).

**Fig. 2:**
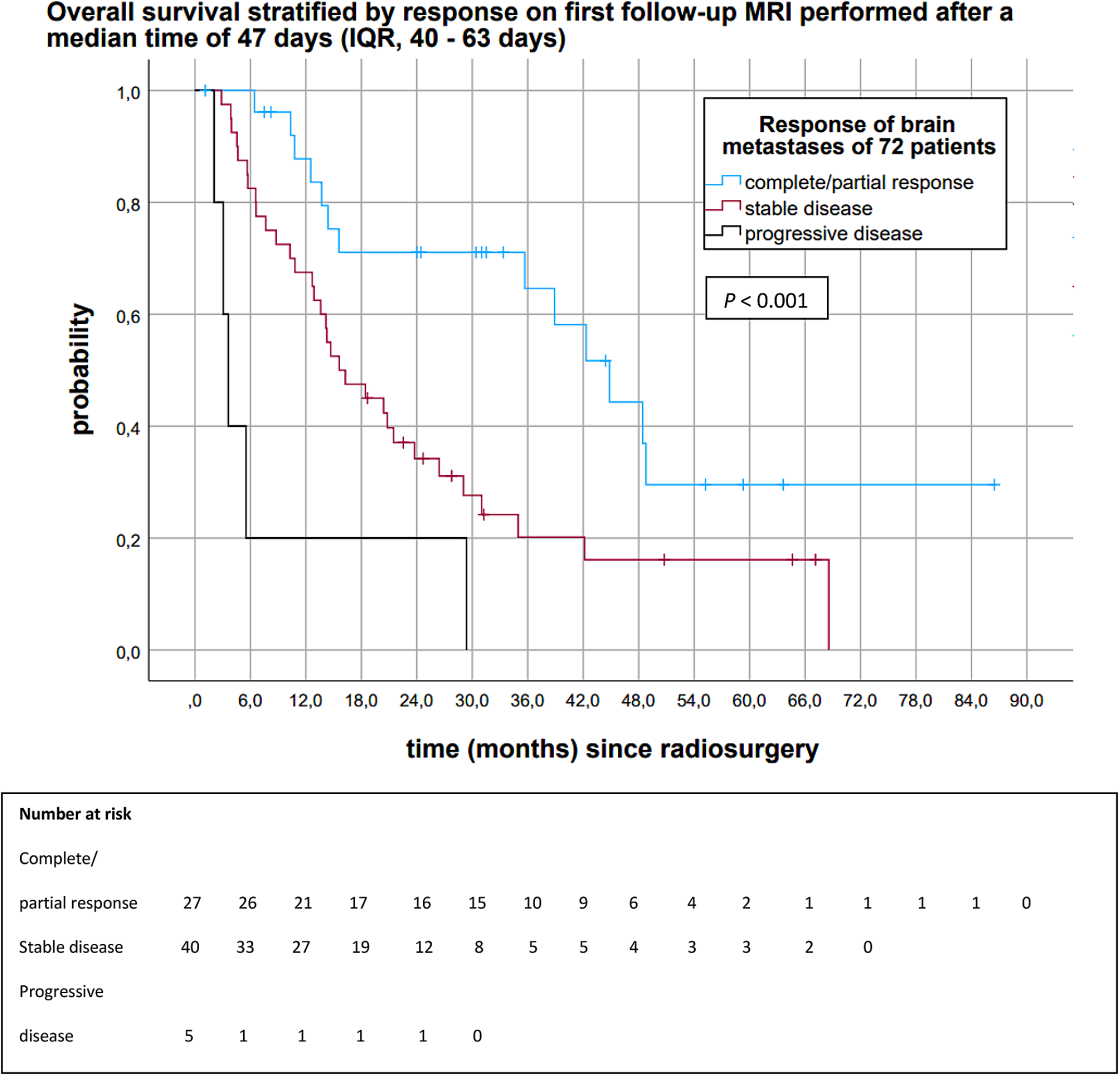
Kaplan-Meier analysis of overall survival stratified by response on 1st follow-up MRIs (*n* = 72)

Figure 2 depicts the Kaplan-Meier analysis of OS stratified by response on 1st follow-up MRIs performed after a median time of 47 days (IQR, 40–63). The median OS of patients with complete/partial response on 1st follow-up MRI was 44.8 months (95%CI 34.6, 55.0) (Fig. 2). The median OS of patients with stable disease and progressive disease on 1st follow-up MRI were 15.6 months (95%CI 9.1, 22.1) and 3.6 months (95%CI 2.4, 4.8), respectively (*P* < 0.001). Patients with complete/partial response of brain metastases on 1st follow-up MRI had 6-, 12-, 18-, 24- and 30-month OS rates of 100%, 87.8%, 71.1%, 71.1%, and 71.1%. Patients with stable disease on 1st follow-up MRI had a 6-, 12-, 18-, 24- and 30-month OS of 82.5%, 67.5%, 47.5%, 34.2%, and 27.6%. Patients with progressive disease had a 6-month OS of 20.0%.

## Local progression-free survival

**Fig. 3:**
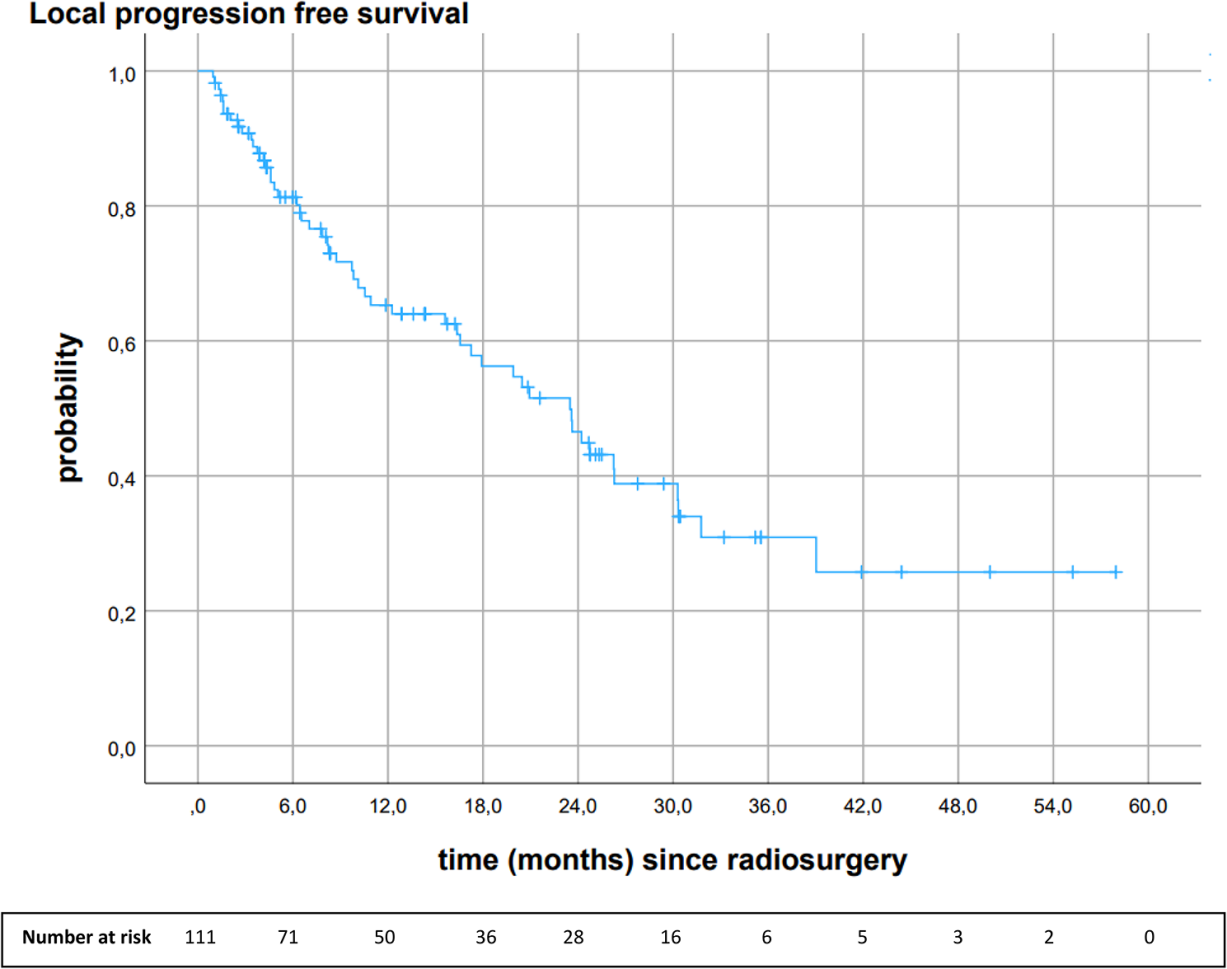
Kaplan-Meier analysis of local progression-free survival of all brain metastases after 18 Gy stereotactic radiosurgery

The median LPFS of all brain metastases was 23.5 months (95%CI 17.2, 29.8). Local progression-free survival rates at 6-, 12-, 18-, 24- and 30 months were 81.3%, 65.3%, 56.3%, 46.5%, and 38.8%. At the end of the follow-up period, 46.8% (n = 52) of brain metastases relapsed, 23.4% (n = 26) showed complete response, 20.7% (n = 23) stable disease, and 9.0% (n = 10) partial response (see Fig. 3).

**Fig. 4:**
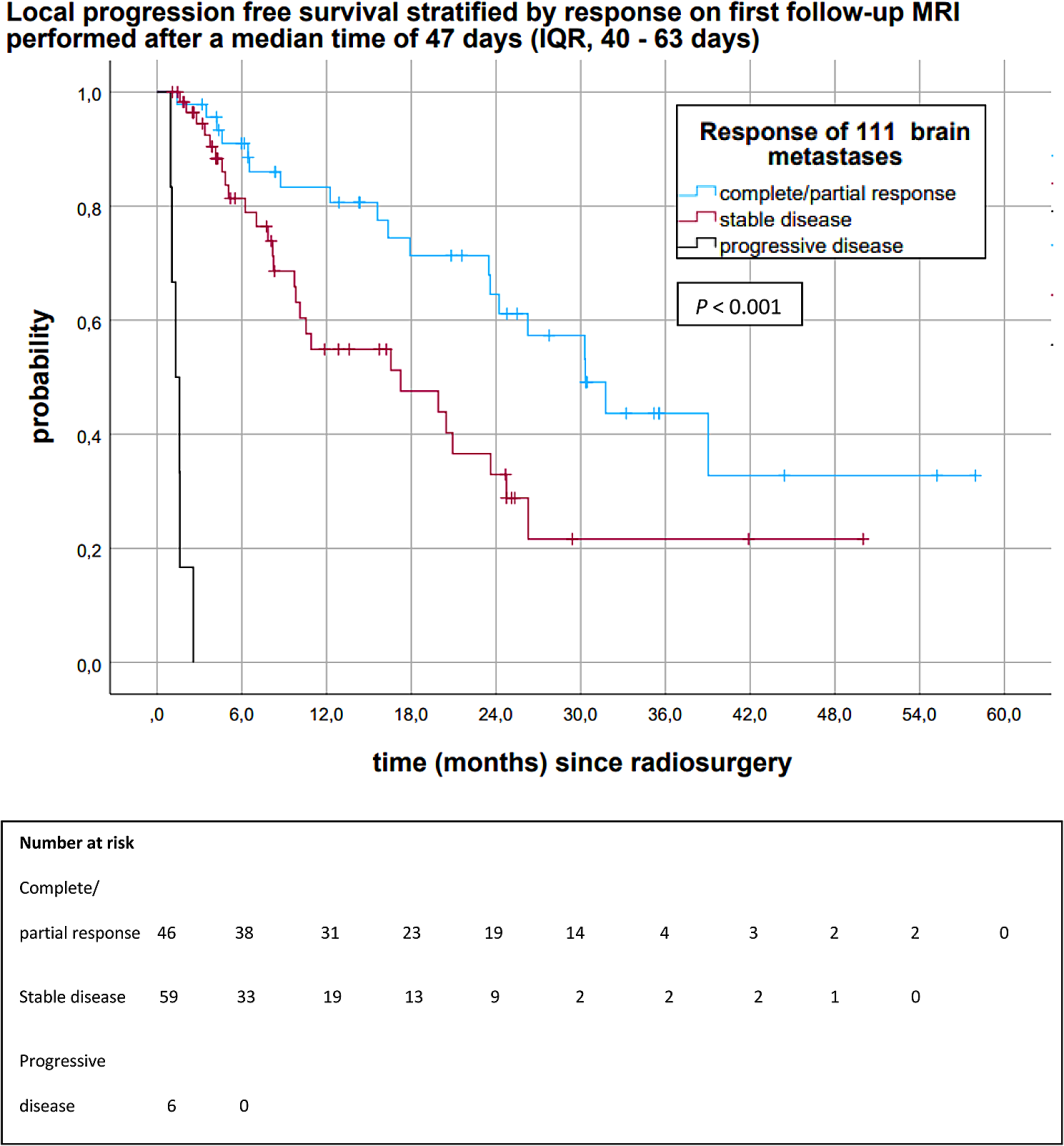
Kaplan-Meier analysis of LPFS of all brain metastases after 18 Gy stereotactic radiosurgery stratified by response on 1st follow-up MRI

Figure 4 shows the Kaplan-Meier analysis of LPFS of all brain metastases stratified by response on 1st follow-up MRIs, which were performed after a median time of 47 days (IQR, 40–63). Median LPFS of brain metastases with complete/partial response on 1st follow-up MRI was 30.3 months (95%CI 23.0, 37.6). Median LPFS of brain metastases with stable disease and progressive disease on 1st follow-up MRI were 17.2 months (95%CI 6.0, 28.4) and 1.3 months (95%CI 0.7, 1.9), respectively (*P* < 0.001). Brain metastases showing complete/partial response on 1st follow-up MRI had a 6-, 12-, 18-, 24- and 30-month LPFS of 91.0%, 83.3%, 71.3%, 64.5%, and 57.3%. Brain metastases with stable disease on 1st follow-up MRI had a 6-, 12-, 18-, 24-, and 30 month LPFS of 81.4%, 54.9%, 47.6%, 32.9%, and 21.6% (Fig. 4).

**Fig. 5:**
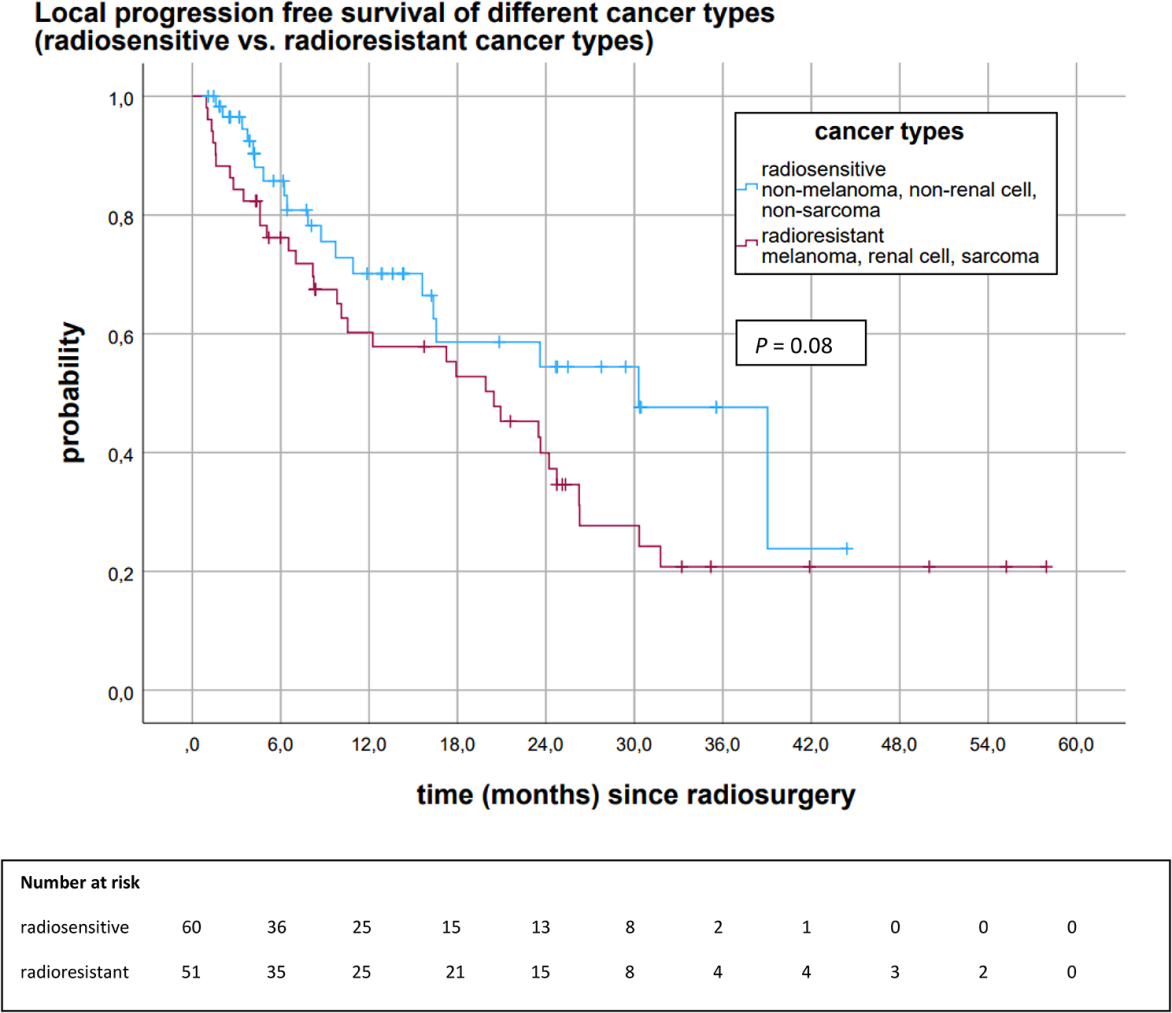
Kaplan-Meier analysis of LPFS of brain metastases stratified by cancer types (radioresistant vs. radiosensitive cancer types)

The proportion of brain metastases with radioresistant histology (malignant melanoma, renal cell cancer, sarcoma) was relatively high (n = 51). A separate analysis addressed the differences in local control stratified by radiosensitivity (malignant melanoma, renal cell cancer, and sarcoma vs. other histology) (Fig. 5). Median LPFS of brain metastases with radioresistant histology was 20.5 months (95%CI 13.2, 27.7). Brain metastases with other/radiosensitive histology (n = 60) had a median LPFS of 30.3 months (95%CI 17.6, 43.0) (*P* = 0.084). Figure 5 depicts the differences in local control. Twelve-, 18-, and 24-month LPFS of brain metastases with radioresistant histology was 60.2%, 52.8%, and 39.9%. Brain metastases with other/more radiosensitive histology had a 12-, 18-, and 24-month LPFS of 70.1%, 58.6%, and 54.4%.

## Distant progression-free survival

Median DPFS was 6.8 months (95%CI 2.1, 11.5). Six, 12-, 18-, 24- and 30-months DPFS rates were 50.3%, 37.8%, 35.7%, 33.1% and 29.8%. In total, 62.5% (n = 45) of patients had brain distant relapse. Most patients (55.6%, n = 25) received another course of stereotactic radiotherapy. Eleven patients (24.4%) received WBRT. Fore patients (8.9%) were treated with immunotherapy or tyrosine kinase inhibitors. Fore patients (8.9%) received best supportive care, and one patient (2.2%) surgery of the metastasis.

## Brain radiation necrosis

Eight brain metastases (7.2%) developed brain radiation necrosis after a median time of 18.4 months (IQR, 9.4–26.5). Two patients complained about neurological symptoms, the remaining patients were symptom-free. Seven brain metastases were resected without evidence of tumor cells. One brain metastasis showed graphically signs of brain radiation necrosis which, however, responded successfully to therapy with bevacizumab. None of the patients died from radiation necrosis.

## Univariate and multivariate analysis of OS and LPFS after stereotactic radiosurgery

Results of the univariate and multivariate analysis of OS are shown in Table 2. In multivariate analysis, KPS was predictive for OS (HR 0.946., 95%CI 0.902, 0.993; *P* = 0.024).

**Table 2:** Univariate and multivariable analysis of overall survival after 18 Gy stereotactic radiosurgery

Characteristics	Univariate model			Multivariate model		
	HR	95%CI	*P*-value	HR	95%CI	*P*-value
**Gross tumor volume**						
≤ 0.3 cm³ (reference)			0.616			0.319
> 0.3 cm³	1.153	0.660, 2.015		1.384	0.730, 2.623	
**Extracranial metastases**						
No (reference)			**0.007**			0.157
Yes	2.411	1.272, 4.567		2.068	0.756, 5.654	
**Control of primary cancer**						
No control (reference)			0.086			0.158
Control	0.611	0.348, 1.071		0.618	0.317,1.205	
Systemic treatment * 3 months before/after SRS						
No (reference)			0.691			0.374
Yes	0.826	0.323, 2.114		0.611	0.206, 1.811	
Karnofsky performance score	0.950	0.923, 0.978	**< 0.001**	0.946	0.902, 0.993	**0.024**
Number of brain metastases	1.257	1.002, 1.577	**0.048**	1.187	0.906, 1.555	0.214
Patient age	1.019	0.994, 1.045	0.138	1.005	0.977, 1.034	0.723
Recursive partitioning analysis (RPA)	1.485	0.913, 2.418	0.111	0.915	0.445, 1.885	0.811
Diagnosis-specific graded prognostic assessment (ds-GPA)	0.517	0.365, 0.732	**< 0.001**	1.023	0.543, 1.926	0.944
**Histology**						
Other (reference)			0.288			0.609
Malignant melanoma/renal cell cancer/sarcoma	0.729	0.408, 1.306		0.828	0.402, 1.707	
**Cerebral progression outside the planning target volume**						
No (reference)			0.119			0.534
Yes	1.658	0.878, 3.130		1.285	0.583, 2.831	

*systemic treatment, chemotherapy, immunotherapy, targeted therapy or anti-hormonal therapy; *SRS*, stereotactic radiosurgery

Table 3 shows the results of the univariate and multivariate analysis of LPFS. In multivariate analysis, a GTV > 0.3 cm³ negatively affected LPFS (HR 2.229, 95%CI 1.172, 4.239; *P* = 0.015). Melanoma, renal cell cancers, and sarcoma had a lower chance of LPFS in comparison to other cancer types (HR 2.330, 95% CI 1.155, 4.699; *P* = 0.018).

**Table 3:** Univariate and multivariate model of local progression-free survival after 18 Gy stereotactic radiosurgery

Characteristics	Univariate model			Multivariate model		
	HR	95%CI	*P*-value	HR	95%CI	*P*-value
**Gross tumor volume**						
≤ 0.3 cm³ (reference)			0.430			**0.015**
> 0.3 cm³	1.249	0.720, 2.166		2.229	1.172, 4.239	
**Extracranial metastases**						
No (reference)			0.360			0.828
Yes	1.318	0.730, 2.379		1.107	0.442, 2.771	
**Control of primary cancer**						
No control (reference)			0.784			0.105
Control	0.924	0.524, 1.628		0.581	0.301, 1.119	
Karnofsky performance score	0.972	0.940, 1.004	0.089	0.976	0.927, 1.028	0.359
Systemic treatment * 3 months before/after SRS						
No (reference)			0.101			0.073
Yes	0.481	0.201, 1.154		0.385	0.136, 1.094	
Number of brain metastases	1.035	0.839, 1.276	0.750	0.926	0.719, 1.192	0.550
Patient age	0.992	0.973, 1.011	0.414	0.987	0.963, 1.011	0.295
Recursive partitioning analysis (RPA)	0.926	0.559, 1.534	0.764	0.701	0.359, 1.366	0.297
Diagnosis-specific graded prognostic assessment (ds-GPA)	0.800	0.583, 1.098	0.166	0.678	0.365, 1.258	0.218
**Histology**						
Other (reference)			0.084			**0.018**
Malignant melanoma/renal cell cancer/sarcoma	1.648	0.935, 2.905		2.330	1.155, 4.699	
**Cerebral progression outside the planning target volume**						
No (reference)			0.699		0.397, 1.910	0.729
Yes	1.132	0.603, 2.127		0.870		

*systemic treatment, chemotherapy, immunotherapy, targeted therapy or anti-hormonal therapy; *SRS*, stereotactic radiosurgery

## Discussion

This retrospective study analyzed our single-institutional results of SRS of comparatively small brain metastases (median GTV 0.30 cm³). Following institutional guidelines and a risk-based selection, patients received 18 Gy SRS instead of 6 × 5 Gy FSRT according to tumor- and patient-related factors (tumor size, location, and adjacent organs at risk). The literature confirms the common practice of using SRS for smaller brain metastases and FSRT in larger ones [9]. A benefit is assumed with FSRT instead of SRS, especially in large and medium-sized brain metastases. The benefit of FSRT in large-sized brain metastases was demonstrated by Minnitti et al. [12] comparing 15 to 18 Gy single-fraction SRS (median GTV 8.8 cm³) with 3 × 9 Gy FSRT (median GTV 12.5 cm³) in brain metastases > 2.0 cm. The most common histology was NSCLC (41% and 42%), while only 15% and 13% were malignant melanoma. The propensity score matching analysis (matching by e.g., tumor size and histology) demonstrated that FSRT was associated with significantly higher 1-year cumulative local control rates (91% vs. 76%) in comparison to SRS, and lower 1-year cumulative incidence rates of radionecrosis (8% vs. 20%). In particular, brain metastases ≥ 3 cm receiving SRS seemed to be predictive of local failure [12]. Chon et al. [22] compared SRS (median 20 Gy) and FSRT (median 35 Gy in 5 fractions) for brain metastases of 2.5–3.0 cm in diameter. The most common primary cancers were lung cancers (53.7% and 52.6%). One-year local control was markedly higher in the FSRT group (92.4%) compared to the SRS group (66.6%). Radiation necrosis was significantly higher in the SRS group than in the FSRT group (HR 8.479, 95%CI 1.966, 36.570) [22]. A meta-analysis on the comparative analysis of SRS and FSRT in brain metastases > 2 cm in diameter finally found no significant differences in local control [6]. However, 1-year radiation necrosis rates were significantly higher in the SRS group (18.2%) compared to the FSRT group (7.1%) [6]. The SRS group of the present study comprised comparatively small-sized brain metastases (median GTV 0.30 cm³, IQR 0.17–0.61 cm³), making it difficult to compare the results with studies mainly including large-sized brain metastases. Since the size of the brain metastases impacts the results after SRS, we discuss studies examining small brain metastases.

Chang et al. [13] analyzed the results of 135 patients with 153 small brain metastases after 20–24 Gy SRS. Malignant melanoma (29.6%), NSCLC (28.1%), and renal cell cancer (23.7%) were the most common primaries. One-year and 2-year local control rates were 86% and 78% in brain metastases ≤ 1 cm (0.5 cm³) and 56% and 24% in brain metastases > 1 cm (0.5 cm³). The 1- and 2-year local control rates for all brain metastases were 69% and 46%, respectively [13], similar to our results of 18 Gy SRS (1- and 2-year local control rates of 65.3% and 46.5%). Other studies focusing on SRS of small brain metastases are reported by Putz et al. [14] and Fokas et al. [23]. Putz et al. [14] analyzed differences in local control and radiation necrosis of small-sized brain metastases treated with a median SRS dose of 18 Gy or 10 × 4 Gy FSRT. Doses were prescribed to the encompassing 80% isodose. Median metastases volumes were 0.23 cm^3^ (IQR, 0.12–0.50 cm^3^) in the SRS group, and 1.42 cm^3^ (IQR, 0.34–4.41 cm^3^) in the FSRT group. The high proportion of malignant melanoma in the SRS group (55.4%) and FSRT group (32.7%) is worth mentioning. The study revealed a higher 1-year local control after FSRT in comparison to SRS (70.2% vs. 55.6%). It was also evident that the 12-month radionecrosis rate was lower in the FSRT group compared to the SRS group (3.4% vs. 14.8%) [14]. Fokas et al. [23] compared the results of SRS (median dose 20 Gy) and FSRT using two dose schedules (7 × 5 Gy and 10 × 4 Gy). The median tumor volume was 0.87 cm^3^ (range, 0.03–13.4 cm^3^) in the SRS group, 2.04 cm^3^ (range, 0.02–27.5cm^3^) in the 7 × 5 Gy group, and 5.93 cm^3^ (range, 0.02–26.8 cm^3^) in the 10 × 4 Gy group. The most common primary cancers were NSCLC in each group (40%, 44%, and 34%) while the proportion of malignant melanoma was low (11%, 5%, and 11%). LPFS was comparable between the three groups. LPFS rates at 1 year were 73% for the SRS group, 75% for the 7 × 5 Gy group, and 71% for the 10 × 4 Gy group. Five patients showed radionecrosis, four of the SRS group, and one of the 7 × 5 Gy group [23].

The literature determines the efficacy of stereotactic radiotherapy using the 1-year local control rate [9]. The systematic review of Wiggenraad et al. [9] showed that the 1-year local control rates varied markedly in single-fraction SRS studies, depending on the single dose. One-year local control rates were > 80% using single doses ≥ 21 Gy, > 60% using single doses ≥ 18 Gy, and < 50% using single doses ≤ 15 Gy. In summary, Wiggenraad et al. recommended a BED 12 of at least 40 Gy, corresponding to a single dose of 20 Gy SRS, to achieve a 1-year local control rate of at least 70% [9]. Our data indicate that brain metastases show a 1-year overall local control rate of 65.3% after 18 Gy SRS, which is within the range of the literature containing high proportions of malignant melanoma. The radio-resistant behavior of metastases of malignant melanoma is well-known in the literature [24]. The present study revealed that brain metastases with radioresistant histology (melanoma, renal cell cancer, and sarcoma) show a 12-month LPFS of 60.2%, whereas brain metastases with other/more radiosensitive histology show a 12-month LPFS of 70.1%. Putz et al. [14] analyzed local control of melanoma brain metastases and non-melanoma brain metastases (10 × 4 Gy FSRT vs. 18 Gy SRS). The study reported a 12-month local control rate of 46.6% in the melanoma group treated with 18 Gy SRS. Non-melanoma histology was associated with a 12-month local control rate of 67.8% in the SRS group. Putz et al. [14] provided evidence that 12-month local control rates were improved in the FSRT arm for melanoma (59.8%) and non-melanoma histology (76.0%) in comparison to the SRS arm. It is a known fact that the radio-sensitivity varies in different phases of the cell cycle. Cells are most resistant in G0 of the cell cycle, a resting phase from which a return to the more radiosensitive cell phases is possible. Therefore, fractionation instead of single-time irradiation, allowing cells from the G0 phase to re-enter the cell cycle, is assumed to be more efficient [10]. We previously published results of FSRT of brain metastases with the fractionation scheme of 6 × 5 Gy [19]. Since it was a risk-based decision to treat small metastases without proximity to critical organs at risk with 18 Gy SRS and larger ones with 6 × 5 Gy FSRT, a direct comparison of the efficacy is not without bias. The risk-based selection according to tumor- and patient-related factors prohibits matched pair analyses. Although we know the bias in the comparative analysis of the two treatment groups, we compared local control after SRS to our results of 103 brain metastases treated with 6 × 5 Gy FSRT. Brain metastases of the SRS group were smaller (median GTV 0.30 cm³) than brain metastases of the FSRT group (median GTV 0.9 cm^3^) [19]. Almost half of the patients had relatively radio-resistant cancers (malignant melanoma, renal cell, or sarcoma) in the SRS group, and 35.6% in the FSRT group. When calculating the BED doses according to the LQ model of Wiggenraad et al. [9], the BED was 41 Gy in the 6 × 5 Gy FSRT group and 36 Gy in the 18 Gy SRS group. The overall 1-year local control was 68.7% in the FSRT group, and 65.3% in the SRS group. The median time to local progression was 23.5 months (95%CI 17.2, 29.8) in the SRS group, while the median has not been reached in the FSRT group. The mean time to local progression was 38.1 months (95%CI 31.4, 44.9) in the FSRT group [19]. Brain radiation necrosis did not appear to be more common in the SRS group in comparison to the FSRT group (7.2% vs. 4.8%). The small size of brain metastases in the SRS group may have contributed to the comparatively low rate of radiation necrosis after SRS. In summary, radiation necrosis depends on several factors (e.g., single-dose [9] in relation to tumor size [4], volume of brain irradiation, BED [9], and additional WBRT [5, 25]), making it difficult to compare different studies.

In summary, difficulties in interpretation arise during analyzing the local control of different studies. Six months local control rates seem to be high in almost all studies irrespective of dose. However, differences in local control are already evident at 1-year follow-up. Explanations may be varying treatment algorithms (e.g., different specification isodoses, dose normalization, target coverage, and safety margins) and wide ranges of biologically effective doses leading to biases regarding local control and radiation necrosis. Finally, definitions of relapse and radiation necrosis were different at many institutions. We should note that many of these studies mentioned have biases by small patient numbers, varying tumor volumes, and cancer types. Due to the more radio-resistant behavior of malignant melanoma, these should be analyzed separately rather than mixed with other cancer types. In addition, some studies included patients who had received simultaneous WBRT improving the local effectivity of SRS [5, 25].

SRS does not prevent distant cerebral failure. In our study, most patients received a second session of stereotactic radiotherapy of new brain metastases. However, we did not analyze the outcome of patients undergoing salvage stereotactic radiotherapy for recurrent brain metastases. Despite the strong evidence supporting the use of SRS, optimal treatment of brain metastases remains controversial because of the lack of prospectively randomized studies of FSRT and SRS. According to radiobiological considerations, FSRT instead of SRS could be associated with advantages in terms of toxicity with the same or even better local control. However, FSRT leads to patient inconvenience as patients have to come back for radiotherapy several times.

## Limitations

This analysis is limited by its retrospective design. We acknowledge that the comparatively small patient numbers reduce the generalizability of the results. The primary strength of the study is the consistent delivery of 18 Gy SRS and the long observation time. Although certain limitations of the present study exist, it provides insight into the stereotactic treatment of brain metastases.

## Conclusion

At present, the choice of stereotactic treatments (SRS vs. FSRT), dose prescription, and dose normalization seems to be individual according to the experience of each center, influencing the results. Our data indicate a 1-year local control rate of 70.1% of brain metastases with radiosensitive histology after 18 Gy SRS. In contrast, radioresistant cancer types such as melanoma, sarcoma, and renal cell cancer show comparatively poor local control. Our data suggest that not all brain metastases respond equally to 18 Gy SRS. We conclude that treatment refinements merit exploration to improve local control of brain metastases. Further efforts are needed to implement a standardized prescribing and reporting of doses guiding the stereotactic treatment of brain metastases.

## Data Availability

The datasets generated and/or analyzed during the current study are not publicly available due to privacy but are available from the corresponding author on reasonable request.

## List of abbreviations

SRS: stereotactic radiosurgery
FSRT: fractionated stereotactic radiotherapy
LPFS: local progression-free survival
OS: overall survival
MRI: magnetic resonance imaging
IQR: interquartile range
GTV: gross tumor volume
WBRT: whole brain radiotherapy
RTOG: Radiation Therapy Oncology Group
LQ model: linear-quadratic model
PTV: planning target volume
KPS: Karnofsky performance score
RPA: recursive partitioning analysis
ds-GPA: diagnosis-specific graded prognostic assessment
DPFS: distant progression-free survival
CT: computed tomogram
MV: megavoltage
BED: biologically effective dose
RANO-BM: Response Assessment in Neuro-Oncology Brain Metastases
PET: positron emission tomography
HR: hazard ratio
CI: confidence intervals
SD: standard deviation
NSCLC: non-small cell lung cancer
